# Effects of pre-storage leukoreduction on erythrocyte concentrates and performance of newer generation leuko-filters at a tertiary care oncology hospital in Western India

**DOI:** 10.4102/ajlm.v14i1.2723

**Published:** 2025-06-25

**Authors:** Deep Madkaiker, Shashank Ojha, Arunkumar N, Kalpesh Chawan

**Affiliations:** 1 Department of Transfusion Medicine, Advanced Centre for Treatment, Research and Education in Cancer, Tata Memorial Centre, Homi Bhabha National Institute, Mumbai, India; 2 Department of Immunohematology and Blood Transfusion, Kasturba Medical College, Manipal, Manipal Academy of Higher Education, Manipal, Karnataka, India

**Keywords:** leukoreduction, red cell indices, quality control, whole blood, leuko-filter, red cell concentrate

## Abstract

**Background:**

Leukoreduction is a post-processing technique that reduces residual leukocytes in cellular blood components. Previous studies have evaluated these parameters mainly among older generation leuko-filters.

**Objective:**

This study evaluated the immediate effects of pre-storage leukoreduction on red cell indices and the performance efficacy of two newer generation leuko-filters.

**Methods:**

This retrospective analysis collected quality control data before and after leukoreduction for erythrocyte concentrates (ECs) from laboratory registers from the Blood Transfusion Laboratory at the Advanced Centre for Treatment, Research and Education in Cancer in Mumbai, India, for the period January 2015 to December 2019. Data related to red cell indices and performance characteristics for Fresenius and Macopharma filters were included.

**Results:**

A total of 500 records was included in the study. All EC units demonstrated a 99.99% leukocyte log reduction, with both filters showing equal efficacy. Post-leukoreduction haemoglobin concentrations were lower than the pre-leukoreduction for all units (*p* < 0.001). Of those prepared from 350 mL units, 11.6% (28/240) had haemoglobin levels under 40 g/bag as compared to 1.1% (3/260) among those prepared from 450 mL units. All indices exhibited statistically significant changes after leukoreduction (*p* < 0.001) except for mean corpuscular haemoglobin (*p* = 0.215). The Fresenius filter required less time for leukoreduction compared to the Macopharma filter (*p* < 0.001).

**Conclusion:**

Red cell indices show several changes following leukoreduction. Further studies are needed to assess the microscopic and functional impact of leukoreduction. Leuko-filters vary in their performance characteristics, which may influence vendor selection.

**What this study adds:**

This study found changes among several red cell indices after leukoreduction of ECs, which have not been extensively studied in previous literature. Further, we found that the newer generation leuko-filters differ in specific performance characteristics, which may influence vendor selection.

## Introduction

The field of transfusion medicine has experienced several challenging periods, which have culminated in the introduction of newer technological advancements to improve the safety of allogenic blood. Leukoreduction was one such paradigm shift in transfusion practices which gained momentum in the 1990s in response to the Variant Creutzfeldt-Jakob disease outbreak.^1^ This resulted in the implementation of universal leukoreduction in the United Kingdom as a risk reduction measure. Universal leukoreduction was also implemented by France to counter the potential spread of transmissible spongiform encephalopathies by donor leukocytes, and, during a general reorganisation of transfusion services, in Canada.^2^

Residual viable donor leukocytes are present in cellular blood components and have been implicated in several complications such as febrile, non-haemolytic transfusion reactions; human leukocyte antigen alloimmunisation and subsequent platelet transfusion refractoriness; transfusion related immuno-modulation; and transmission of infectious leukotropic agents such as cytomegalovirus and human T-lymphotropic virus I/II, and transfusion-associated graft-versus-host disease.^3^ Leukocyte depletion is known to reduce transfusion-related immunomodulation effect in patients, which depends on the degree of leukoreduction, with leuko-filtration being far more effective than buffy coat depletion.^4^ Additionally, white blood cells (WBCs) are known to damage red cells, which further exacerbates red cell storage lesions during their storage.^5^

Presently, leukoreduction is achieved via leuko-filters, comprising an external plastic housing which fits tightly around the filtration medium.^2^ The current generation of leuko-filters are highly efficient, as they have an excellent leukocyte removal efficacy and are able to achieve a 3–4 log reduction (99.9% – 99.99%) as compared to 1st and 2nd generation filters (90% – 96%).^6^ Barrier filtration and cell adhesion are the major contributing mechanisms leading to a very high efficacy of leukocyte removal. Most previous studies on leukoreduction filters have focused on evaluating erythrocyte concentrate (EC) quality parameters and filter performance characteristics, primarily among older generations of leukoreduction filters.^7,8^ Fewer studies have reported changes in the red cell indices.^5,9,10^

Blood transfusion services in India are decentralised, with varying practices across the country. Leukoreduction is not a mandatory requirement in India. However, it may be implemented by the blood centre based on selective patient profiles. At our tertiary care oncology setup at the Department of Transfusion Medicine at the Advanced Centre for Treatment, Research and Education in Cancer in Mumbai, India, we cater to all types of oncology patients, and leukoreduced blood components are specifically indicated for haemato-oncology patients, since they are at risk of human leukocyte antigen alloimmunisation and future risk of poor transplant outcomes. In our current study, we assessed the immediate effects of pre-storage leukoreduction on red cell indices of ECs, and the performance efficacy of two newer-generation leuko-filters available at our centre.

## Methods

### Ethical considerations

This study was approved by the Institutional Ethics Committee-III at the Advanced Centre for Treatment, Research and Education in Cancer, Mumbai, India (Project number 900713 and Institutional Ethics Committee approval number 566/2020 dated 19 October 2020). Waiver of consent was approved for this study, since it involved technical anonymised data (not involving human or animal research) delinked from original blood donor records, not available in the public domain. These quality control (QC) records were maintained as hard copies, which were recorded in laboratory registers, and the complete blood count (CBC) printouts from the haematology analyser, which were filed and stored in locked cabinet sections in the component laboratory. Furthermore, access to these documents was restricted to the respective section staff, technical supervisors, and transfusion laboratory in-charge personnel.

### Study design and setting

This retrospective analysis of leukoreduction QC records for ECs recorded from 01 January 2015 to 31 December 2019 was conducted at the Department of Transfusion Medicine (Blood Transfusion Laboratory) at the Advanced Centre for Treatment, Research and Education in Cancer, Mumbai, India. This hospital serves as a tertiary care oncology hospital catering to all types of cancer patients, including haemato-oncology. All EC units subjected to leukoreduction are prepared from whole blood which has been collected from voluntary blood donors in accordance with the national blood donor selection guidelines approved by the Directorate General of Health Services, India, which is the regulatory authority for blood safety in India. Quality control is mandated by the Indian regulatory authorities for 1% of all leukoreduced ECs prepared from 350 mL or 450 mL whole blood. Quality control is performed as a routine measure in the laboratory, to ensure the quality of blood components and that leukoreduction was performed by trained and competent staff as per standard operating procedures in the department of transfusion medicine, and documented in the respective QC registers.

### Inclusion/exclusion criteria

Erythrocyte concentrates undergoing leukoreduction between 01 January 2015 and 31 December 2019 were included in the study, whereas ECs not subjected to leukoreduction, QC records with missing data, and units that were leukoreduced outside designated 5-year study period were excluded from the study.

### Sample and data collection

Representative samples were collected from the tubing of the EC unit. The CBC samples were collected in ethylenediaminetetraacetic acid vacutainers (BD Sciences, Mumbai, Maharashtra, India) and were analysed using the Siemens ADVIA^®^ 2120i (Siemens AG, Erlangen, Germany), a 5-part differential haematology analyser. These ethylenediaminetetraacetic acid samples were tested immediately and subsequently discarded as per national biomedical waste management guidelines. Quality control data, including filter performance data for leukoreduced ECs, were recorded manually in physical laboratory registers maintained in Department of Transfusion Medicine. Red cell indices were recorded from the CBC report printouts from the Siemens ADVIA^®^ analyser.

### Laboratory analyses

The data obtained from the QC records were CBC parameters of pre-leukoreduction and post-leukoreduction samples from red cell concentrates, namely WBC, red blood cell count (RBC), haemoglobin, haematocrit, mean corpuscular volume (MCV), mean corpuscular haemoglobin (MCH), mean corpuscular haemoglobin concentration, cellular haemoglobin, and platelet count. Post-leukoreduction WBC count was measured using Nageotte’s haemocytometer (Paul Marienfeld GmbH & Co. KG, Lauda-Königshofen, Germany) as per validated departmental standard operating procedures, by trained technical staff. Demographic data related to donors were not captured in the QC laboratory registers; only the date of collection and donor unit numbers were recorded, along with other QC data, while the actual donor records were stored separately. The two newer generation leuko-filters (Macopharma Leucolab LCG2b: Macopharma, Tourcoing, France; Fresenius BioR: Fresenius Kabi AG, Homburg, Germany) used at our centre were also assessed for their performance efficacy using the following parameters: WBC log reduction, hold-up volume of the filter, and time taken for filtration. These leuko-filter performance characteristics were also recorded in the QC laboratory registers.

### Data analysis

Data were captured from the laboratory registers in a case record form, checked by the investigators during entry and cleaned using Microsoft Excel (Redmond, Washington, United States). Data were analysed using IBM SPSS Statistics for Windows, Version 24.0 (IBM Corp., Armonk, New York, United States). Data were descriptively summarised using median and interquartile range (IQR) for continuous data, while categorical data were reported as frequency and percentage. Normality assumptions were tested using Shapiro-Wilk’s test. The pre- and post- outcomes in the study were compared using the paired Wilcoxon signed rank test for pre-leukoreduction and post-leukoreduction comparisons of red cell indices, and the Mann *U* Whitney test for inter-filter comparisons, depending on the normality of the data. The level of significance for all statistical analysis was established at *p* < 0.05.

## Results

A total of 500 leukoreduction QC records for ECs were reviewed during the study period, with 34 QC records with missing CBC data excluded from the final analysis. Of these, 52% (260/500) were derived from 450 mL whole blood donations (R450), while the rest were prepared from 350 mL whole blood donations (R350). The majority, 69.2% (346/500), of ECs were filtered using Macopharma Leucolab LCG2b, with the remaining 30.8% (154/500) filtered using the Fresenius BioR leuko-filter. Among the ECs filtered with the Macopharma filter, 53.8% (186/346) were R450 units, while the rest were R350. The Fresenius BioR filter was used for almost an equal number of ECs units prepared from 350 mL (74/154; 48.1%) and 450 mL (80/154; 51.9%) whole blood donations.

### Comparison of pre-leukocyte reduction and post-leukocyte reduction red cell indices

There were no instances of leukoreduction failure, even with a wide range of pre-leukoreduction WBC loads (12.4 × 10^3^/uL – 0.6 × 10^3^/uL) in the EC units. Additionally, the post-leukoreduction units showed a mean reduction in platelet count of approximately 97.2% ± 6.0%.

In terms of haemoglobin concentration, a statistically significant reduction was seen in the post-leukoreduction units (*p* < 0.001). The difference in pre-leukoreduction haemoglobin content of the R350 units compared to the R450 units was statistically significant (R350 [median = 54.9 g/bag, IQR = 50.3–63.7] vs R450 [median = 62.5 g/bag, IQR = 58–70], *p* < 0.001). Post-leukoreduction haemoglobin concentrations were statistically significant and lower than the pre-leukoreduction haemoglobin levels for all EC units (*p* < 0.001). Post-leukoreduction haemoglobin concentrations were also noted to be lower and statistically significant in the R350 units compared to the R450 units (R350 [median = 47.5 g/bag, IQR = 42.8–56.8] vs R450 [median = 55.3 g/bag, IQR = 51.1–61.1], *p* < 0.001).

Despite all units meeting the QC criteria for leukoreduced red cell concentrates in India, we found that 11.6% of R350 units (28/240) had haemoglobin levels under 40 g/bag (as per European and United Kingdom guidelines).^11^ Meanwhile, only 1.1% (3/260) of R450 units had haemoglobin levels below this threshold.

All red cell indices exhibited statistically significant changes after leukoreduction (*p* < 0.001), except for MCH (*p* = 0.215) (Table 1).

**TABLE 1:** Red cell indices for pre- and post-leukoreduction samples, at the Advanced Centre for Treatment, Research and Education in Cancer, Mumbai, India, 01 January 2015 to 31 December 2019.

Complete blood count parameters	Pre-leukoreduction values		Post-leukoreduction values		Mean difference	s.e. difference	*p*
	Mean	s.d.	Mean	s.d.			
Red blood cell count (×10^6^/uL)	6.14	1.07	5.91	0.64	0.09	0.05	< 0.001*
Haemoglobin (g/dL)	18.4	2.3	17.5	1.7	0.35	0.12	< 0.001*
Mean corpuscular haemoglobin concentration (g/dL)	30.3	1.3	30.1	1.3	0.2	0.04	< 0.001*
Cellular haemoglobin (g/dL)	28.4	2.3	28.3	2.3	0.1	0.06	< 0.001*
Platelet count (×10^3^/uL)	122	84.3	2.42	4.97	109	3.77	< 0.001*
Haematocrit (%)	58.6	7.1	55.9	5.3	0.95	0.35	< 0.001*
Mean corpuscular volume (fL)	93.7	7.15	96.1	37.7	−0.45	1.67	< 0.001*
Mean corpuscular haemoglobin (pg)	29.9	4.73	29.8	3.82	−0.04	0.23	0.215

s.d., standard deviation; s.e., standard error.

* , *p* < 0.05 was considered significant according to the paired Wilcoxon signed rank test.

**FIGURE 1:**
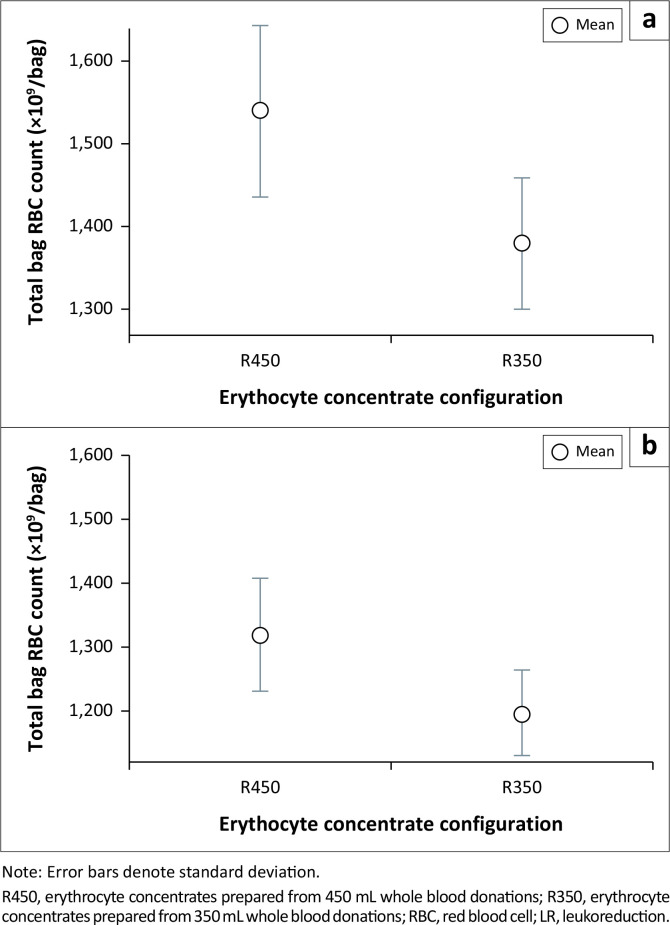
Pre-leukoreduction and post-leukoreduction distribution of haemoglobin, haematocrit and red blood cell counts of erythrocyte concentrates, at the Advanced Centre for Treatment, Research and Education in Cancer, Mumbai, India, 01 January 2015 to 31 December 2019.

Among the remaining parameters, all indices showed a statistically significant decrease (*p* < 0.001), except for MCV, which showed a statistically significant increase (*p* < 0.001). However, the absolute magnitude of difference between the pre-leukoreduction and post-leukoreduction for all red cell indices was found to be small. The most affected haematological parameters were haemoglobin, RBC, and haematocrit, with haematocrit showing a considerable narrowing of its range after leukoreduction (Figure 1).

### Total red blood cell content in erythrocyte concentrate units (red blood cell count × volume of erythrocyte concentrate)

**FIGURE 2:**
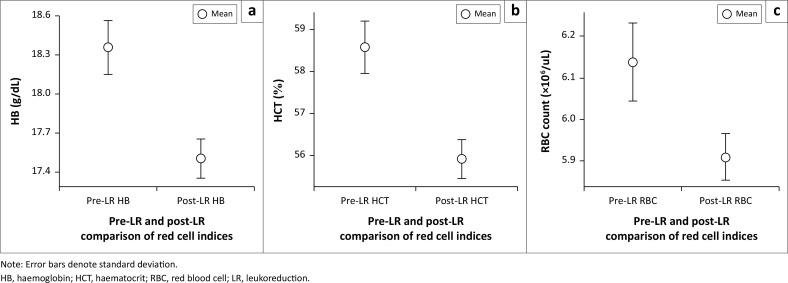
Distribution of a) pre-leukoreduction and b) post-leukoreduction, total red blood cell content in bag for the two erythrocyte concentrate configurations (R450 and R350), at the Advanced Centre for Treatment, Research and Education in Cancer, Mumbai, India, 01 January 2015 to 31 December 2019.

The total RBC content was higher in all pre-leukoreduction units (R350 and R450) than the post-leukoreduction units (Figure 2). Another notable finding was the narrower range of red cell count in the post-leukoreduction ECs. Furthermore, as expected, mean total RBC counts in R450 units were higher than those in R350 units, both before and after leukoreduction.

Both filters demonstrated an equal efficacy in obtaining a 3–4 log WBC reduction post-filtration, with all units in the study meeting the leukoreduction criteria of < 5 × 10^6^ WBCs per bag. Nevertheless, only 33.4% (167/500) of the units achieved a stricter leukoreduction target of < 1 × 10^6^ WBCs per bag. Erythrocyte concentrates filtered using the Macopharma filter comprised a higher proportion of such units than those filtered using the Fresenius filter. Among those filtered using the Macopharma filter, 76 out of 186 were derived from 450 mL whole blood and 56 out of 160 from 350 mL whole blood. Whereas for those ECs filtered using the Fresenius filter, 17 out of 80 were prepared from 450 mL whole blood and 18 out of 74 from 450 mL whole blood WB, which failed to meet this stricter criterion.

### Leuko-filter data

The lower red cell recovery observed in the post-filtration units is attributable to the hold-up volume retained in the filter housing medium.

**FIGURE 3:**
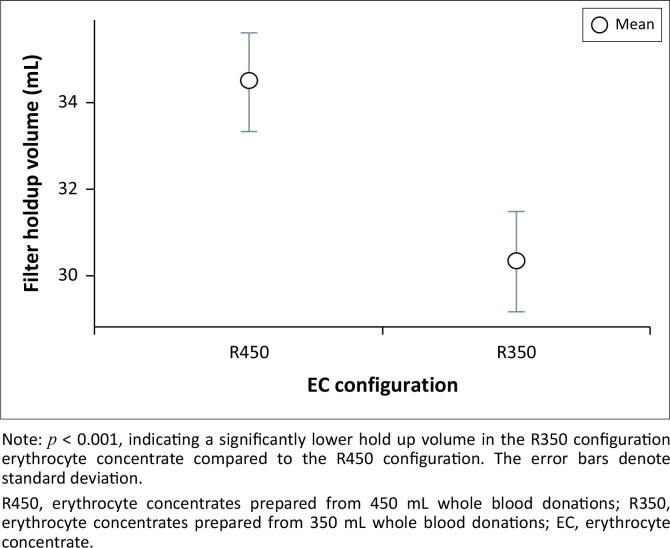
Distribution of filter hold-up volumes for the two erythrocyte concentrate configurations (R450 and R350), at the Advanced Centre for Treatment, Research and Education in Cancer, Mumbai, India, 01 January 2015 to 31 December 2019.

Although no statistically significant difference was noted between the two manufacturers (*p* = 0.88 for R350 units and *p* = 0.193 for R450 units), there was a statistically significant lower hold-up volume in the R350 units compared to the R450 units, *p* < 0.001 (Table 2, Figure 3).

**TABLE 2:** Summary of leuko-filter performance characteristics, at the Advanced Centre for Treatment, Research and Education in Cancer, Mumbai, India, 01 January 2015 to 31 December 2019.

Filter/Configuration	Macopharma (*N* = 346)		Fresenius (*N* = 154)		*p*
	Mean	s.d.	Mean	s.d.	
**Hold-up volume (mL)**					
R350	30	8.16	30.9	10.7	0.88
R450	33.9	8.55	36	11.5	0.193
**Time taken for filtration (min)**					
R350	20.9	5.52	13.4	3.58	< 0.001
R450	20.1	5.61	15.9	4.98	< 0.001
**Total residual bag WBC (× 10** ^**6**^ **/bag)**					
R350	1.3	1.4	0.88	1.2	0.062
R450	1.9	2.9	1.4	1.6	< 0.001
**Pre-leukoreduction volume (mL)**					
R350	323	40.7	290	16.3	0.067
R450	355	37.6	365	24.7	< 0.001
**Post-leukoreduction volume (mL)**					
R350	293	42.2	259	19.5	0.09
R450	321	37.5	329	26.5	< 0.001
**Pre-leukoreduction total bag RBC (× 10** ^**9**^ **/bag)**					
R350	1280	2.15	1577	1.97	< 0.001
R450	1421	2.03	1837	2.26	0.004
**Post-leukoreduction total bag RBC (× 10** ^**9**^ **/bag)**					
R350	1136	2.07	1320	1.76	0.007
R450	1246	2.05	1503	1.87	0.060

Note: Volume of whole blood donations that was used for preparation of leukoreduced erythrocyte concentrates: Macopharma (350 mL [*n* = 160], 450 mL [*n* = 186], *p* < 0.001); Fresenius (350 mL [*n* = 80], 450 mL [*n* = 74], *p* < 0.001).

R450, erythrocyte concentrates prepared from 450 mL whole blood donations; R350, erythrocyte concentrates prepared from 350 mL whole blood donations; RBC, red blood cell count; s.d., standard deviation; WBC, white blood cells.

**FIGURE 4:**
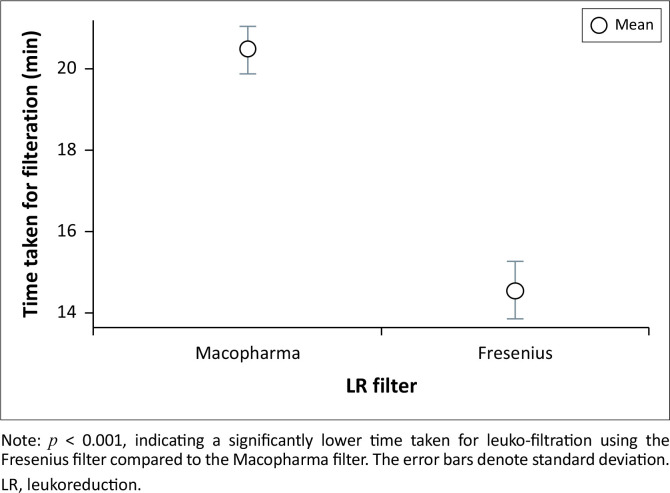
Distribution of leuko-filtration time for the two different manufacturers of leuko-filters, at the Advanced Centre for Treatment, Research and Education in Cancer, Mumbai, India, 01 January 2015 to 31 December 2019.

The difference in time taken for filtration of R350 and R450 units was not found to be statistically significant (*p* = 0.343). In contrast, inter-filter comparisons revealed that ECs undergoing leukoreduction with the Fresenius filter required statistically significantly less time compared to those processed with the Macopharma filter, *p* < 0.001 (Figure 4).

## Discussion

Our study showed that both leuko-filter brands were equally efficacious in achieving leukoreduction to a residual leukocyte count of less than 5 × 10^6^/bag. Post-leukoreduction haemoglobin concentrations were statistically significant and lower in all units as a result of the hold-up volume in the leuko-filter. We noted that 11.6% of R350 units (28/240) had post-leukoreduction haemoglobin levels under 40 g/bag (as per European and United Kingdom guidelines).^11^ Meanwhile, only 1.1% (3/260) of R450 units had haemoglobin levels below this threshold. All red cell indices exhibited statistically significant changes after leukoreduction except for MCH. These changes were noted to be statistically significant and lower, post-leukoreduction, except for MCV which showed a statistically significant increase. Another notable finding was the narrower range of red cell count in the post-leukoreduction ECs. Although no statistically significant difference in hold-up volume was noted between the two manufacturers, there was a statistically significant lower hold-up volume in the R350 units compared to the R450 units. Lastly, the time taken for filtration of R350 and R450 units was not found to be statistically significant. However, in contrast, inter-filter comparisons showed that ECs undergoing leukoreduction with the Fresenius filter required statistically significantly less time compared to those processed with the Macopharma filter.

Our leukoreduction efficacy aligns with previous studies from India published in 2021^12^ and 2022^13^ which showed that the current generation of leuko-filters is highly effective at leukoreduction of red cell concentrates. Two Indian studies from 2014 to 2023 have also reported bag haemoglobin less than 40 g following leukoreduction and it would therefore be advisable that red cells from 450 mL units are allocated for leukoreduction to offset the losses occurring in the filter casing.^14,15^

Among other red cell indices assessed, all indices showed a statistically significant change after leukoreduction, except for MCH. However, the pre-leukoreduction and post-leukoreduction differences for all red cell indices were noted to be small in absolute magnitude. These findings are in accordance with two previous studies – an Indian study by Sonker, Dubey and Chaudhary in 2014,^15^ and an American study by AuBuchon et al. in 1997,^7^ which have reported a decrease in the RBC content, haemoglobin and haematocrit. The increase in MCV found in our study was similar to a Chinese study by Ran et al. in 2011^16^ and the Egyptian study from 2016 by Mahmoud and Hassan.^10^ An Italian study by Pertinhez et al. from 2016 showed two intriguing findings, the first being that non-leukoreduced ECs showed a greater increase in MCV. The other finding was that the increase in MCV exhibits a linear increment when measured for leukoreduced ECs, as compared to non-leukoreduced units. These non-leukoreduced units showed a sigmoidal change in MCV, speculating that leukoreduction may select a homogeneous population of RBCs and support the hypothesis that leuko-depleted RBCs are characterised by a higher deformability that would favour their passage through the filter and are, therefore, more resistant to cell volume impairment during storage.^17^ The beneficial effects of leukoreduction on red cell storage was also reflected in a Canadian study in 2005, wherein leukoreduction was proposed to improve the quality of red cell concentrates by demonstrating less haemolysis than conventional, unfiltered products.^18^

However, Ghezelbash et al.^9^ from Iran in 2017 did not find a statistically significant difference in the MCH of leukoreduced units, while our study found a statistically significant decline in post-leukoreduction MCH. This study further evaluated the changes in red cell indices over the storage duration, showing a decline in MCH and mean corpuscular haemoglobin concentration. Meanwhile, the study by Antonelou et al. from Greece in 2012 had shown an increase in the MCH parameter in both young and senescent red cell units.^5^ The Chinese study by Ran et al. showed that irradiation, filtration, and combined irradiation and filtration in pre-storage units can cause damage in red cells and intensify the RBC storage lesions which could occur in a very short time frame (< 1 day).^16^

Furthermore, our study was comparable to the French study in 1988 by Andreu et al. which showed a similar 1-log platelet reduction in leukoreduced ECs, which might contribute to the beneficial effects of leukoreduction.^8^ However, an American study by Heaton et al. in 1994 reported a much higher platelet reduction in their study, which might be attributable to use of a different leukoreduction filter.^19^ Steneker et al., in their study from The Netherlands in 1993, had shown that the presence of viable platelets may contribute to an improved performance of leukofiltration of the blood components.^20^

The two manufacturers evaluated in our study demonstrated equal efficacy, consistently achieving high levels of leukoreduction, as evidenced by the Indian study by Sonker et al. in 2014 and the American study by Heaton et al. in 1994.^15,19^ While some units (33.4%) did not meet the more stringent Council of Europe guidelines,^11^ the necessity of reducing residual WBCs below 5 × 10^6^/bag lacks substantial evidence, especially considering that complete elimination of residual WBCs is not possible. Attempts to further reduce residual leukocytes to levels < 1 × 10^6^/bag in the absence of confirmatory evidence (which exists for residual counts < 5 × 10^6^/bag) is unlikely to be cost effective, especially in the setting of developing countries.

An inherent drawback of the leuko-filtration process is the hold-up volume retained in the filter housing medium. Our study found that the hold-up volume was statistically significantly higher in ECs filtered by Macopharma filters (10.3% ± 3.5%) compared to Fresenius filters (9.56% ± 2.72%). While most filtered units exhibited post-filtration losses of less than 15%, approximately 4.4% of the units had hold-up volumes exceeding 15% within the leuko-filters. Despite this, all units complied with national QC guidelines, which require 75% of tested units to meet the QC standards. Similar red cell losses in leuko-filters have been reported in previous studies by Andreu et al. in 1988, and AuBuchon et al. from the United States in 1997.^7,8^ We also noted that the hold-up volume was statistically significant and lower in the R350 units compared to R450 units.

Lastly, the inter-filter comparisons also showed a difference in the time taken for leukoreduction, with the Fresenius filters demonstrating higher efficiency with faster filtration rates compared to the Macopharma filter. Leuko-filtration rates documented in published literature exhibit considerable variability, influenced by factors such as filter composition, ambient processing temperature, and the initial specifications of red cells prior to filtration, as shown in the study from The Netherlands by Pietersz, Steneker and Reesink in 1993.^21^ As anticipated, there was a noticeable difference in filtration times between R350 and R450 units, although this difference was not statistically significant. The time taken for filtration depends on the filter-housing medium, and may impact the workload distribution in a large-volume hospital. Along with the cost, this may be a deciding factor when different leuko-filters with equivalent performance characteristics are evaluated for hospital supply.

Our study is the first Indian study to evaluate two newer-generation filters for their performance efficacy and to compare the pre-leukoreduction and post-leukoreduction changes in red cell indices. Our findings support previously published data and also present new data, especially with regard to changes in red cell indices where prior comparative research has been lacking.

### Limitations

The retrospective design of the research study precluded the evaluation of storage duration-related changes in red cell indices. Further, we were only able to include those units which had been subject to a QC check. Donor characteristics such as age, sex and other medical information which are known to influence EC characteristics were not evaluated as part of this study. Additionally, parameters such as supernatant potassium levels, haemoglobin levels, and haemolysis percentage were not investigated. Lastly, patient outcomes following the administration of leukoreduced red cells were not monitored as part of this study.

### Conclusion

Red cell indices show several changes following leukoreduction, but further studies are needed to assess the microscopic and functional impact of leukoreduction.

The newer-generation filters show a good performance efficacy for leukoreduction. Erythrocyte concentrates prepared from 450 mL whole blood units should be preferred for preparation of leukoreduced units subject to local operational constraints, because of the higher red cell recovery. Leuko-filters vary in their performance characteristics such as filtration time and hold-up volume, which may influence vendor selection for hospital purchase.
